# Genotoxic and Toxic Effects of The Flame Retardant Tris(Chloropropyl) Phosphate (TCPP) in Human Lymphocytes, Microalgae and Bacteria

**DOI:** 10.3390/toxics10120736

**Published:** 2022-11-28

**Authors:** Maria Antonopoulou, Dimitris Vlastos, Margarita Dormousoglou, Spyridon Bouras, Maria Varela-Athanasatou, Irene-Eleni Bekakou

**Affiliations:** 1 Department of Sustainable Agriculture (Former Department of Environmental Engineering), University of Patras, 30100 Agrinio, Greece; 2 Department of Biology, Section of Genetics Cell Biology and Development, University of Patras, 26500 Patras, Greece

**Keywords:** tris(chloropropyl) phosphate (TCPP), flame retardants, human lymphocytes, genotoxicity, cytotoxicity, aquatic organisms

## Abstract

Tris(chloropropyl) phosphate (TCPP) is a characteristic and widely used organophosphorus flame retardant. TCPP is comprised of four isomers and the most abundant is tris(1-chloro-2-propyl) phosphate. TCPP can be released into the environment, with potential impacts on living organisms and humans due to its extensive industrial use. Aiming to assess the potential risks of TCPP on human health and the environment, its toxic and genotoxic effects—using organisms from different trophic levels, i.e., bacteria, green microalgae, and human cells—were investigated. TCPP exposure at nominal concentrations of 10, 20, 30 and 40 μg mL^−1^ was studied to identify the potential risk of inducing genotoxic effects in cultured human lymphocytes. Treatment with 30 and 40 μg mL^−1^ of TCPP induced marginally significant micronuclei (MN) frequencies as well as cytotoxic effects. Freshwater microalgae species treated with TCPP (0.5, 1, 10, 20 and 50 μg L^−1^) showed different growth rates over time. All the tested microalgae species were adversely affected after exposure to TCPP during the first 24 h. However, differences among the microalgae species’ sensitivities were observed. In the case of the freshwater species, the most sensitive was found to be *Chlorococcum sp*. The marine algal species *Dunaliella tertiolecta* and *Tisochrysis lutea* were significantly affected after exposure to TCPP. The effects of TCPP on *Aliivibrio fischeri* that were observed can classify this flame retardant as a “harmful” compound. Our results suggest a potential risk to aquatic organisms and humans from the wide utilization of TCPP and its consequent release into the environment. These results highlight that further research should be conducted to investigate the effects of TCPP individually and in combination with other organophosphorus flame retardants in various organisms. In addition, the concern induced by TCPP points out that measures to control the introduction of TCPP into the environment should be taken.

## 1. Introduction

Organophosphates esters (OPEs) are a large class of compounds widely used in industry as flame retardants and plasticizers for a variety of products and applications, i.e., electronics, building materials, vehicles, furniture, car seats, plastics, and fabrics. The widespread and frequent use of OPEs poses a risk to human health, given that they have been detected and quantified in various tissues in the human body [1,2,3,4].

One of the most-used organophosphates, used as a flame retardant, is Tris(chloropropyl) phosphate (TCPP). Commercial TCPP is a mixture of isomers, and its composition may vary. Tris(1-chloro-2-propyl) phosphate is the most used and most frequently detectable isomer in commercial products. Additional isomers that are frequently used include bis(2-chloro-1-methylethyl)-2-chloropropyl phosphate, bis(2-chloropropyl) 2-chloroisopropyl phosphate and tris(2-chloropropyl) phosphate (Table S1) [5].

TCPP is used in the form of a flexible and a rigid polyurethane foam in furniture and construction materials, respectively, as well as in other products such as textiles, paints, coatings, adhesives and electronics. TCPP can be used as a substitute for various brominated as well as other chlorinated flame retardants because of their toxicity concerns [5]. The total consumption of TCPP in 2012 was approximately 54 million pounds (~27,000 tons) [6].

The regulatory status of TCPP in relation to the potential risk of the use of TCPP from 2015 until today can be described as follows: TCPP is listed on the Toxic Substances Control Act (TSCA) Inventory and is under investigation by the U.S. Environmental Protection Agency (EPA) for its impact on consumers, the general population and aquatic organisms. Currently, no regulations restrict the production or use of TCPP in the United States [7]. In Europe, until 2016, TCPP was not registered under the REACH (Registration, Evaluation, Authorization and Restriction of Chemicals) Regulations [8]. In 2018, the European Chemical Agency (ECHA) published an assessment report on chlorinated flame retardants (including TCPP) used in flexible polyurethane (PUR) foams. The evaluation pointed out the risks of children’s exposure to these substances, which are used in the manufacture of children’s items and furniture. Based on the above, the ECHA recommended a restriction proposal under the REACH Regulations; however, this was afterwards “withdrawn” to restrict the use of TCPP in flexible PUR foams in certain products. The ECHA stated that it was expecting new data from the USA on the carcinogenicity of TCPP (US NTP studies—National Toxicology Program) that are not yet available [9,10]. Currently, TCPP is registered under the REACH Regulations, but it is not being manufactured in and/or imported to the European Economic Area. However, that does not prevent the presence of this substance in a variety of imported products used by consumers and/or professional workers at industrial sites and in manufacturing [11].

TCPP can be released into the environment and has potential adverse effects on living organisms and humans due to its extensive industrial use in manufacturing and numerous consumer products [11]. The main source of TCPP in natural water bodies and effluents has been reported to be conventional wastewater treatment plants (WWTPs) [1]. Up to now, TCPP has been found in drinking, ground and surface waters, as well as in soil, sediment, household dust, indoor air and ambient air [12,13,14]. TCPP appears to be recalcitrant in water [15] and it is worth noting that the half-life of TCPP in the environment exceeds 60 days [12].

A recent review reported that TCPP is an environmentally abundant OPE and has been detected at elevated concentrations in indoor and outdoor air, wastewater and surface water in several countries around the world [16]. Rauert et al. (2018) [17] investigated the presence of 18 OPEs at 48 sites in different countries and reported that TCPP is the most frequently detected flame retardant in the outdoor atmosphere, accounting for 45% of the total concentration of detected OPEs.

Human exposure to TCPP can occur through inhalation, oral and/or dermal contact or when working with consumer products containing TCPP. The presence of TCPP in indoor as well as outdoor environments increases its potential effects on human health because of its intensive use as a consumer product. The oral and dermal exposure of children to items containing TCPP (i.e., car safety seats, baby slings, mattresses, etc.) poses a potential health and safety issue that should be always under investigation and control by the regulatory authorities [12,13,18]. The European Commission (2014) [19] stated that TCPP is regarded as a potential carcinogen and suggests restrictions on TCPP use in children’s supplies. According to the findings of Frederiksen et al. (2018) [20], TCPP can easily permeate through the skin.

To our knowledge, there are limited genotoxicity studies on TCPP [21,22], and as stated in a recent NTP’s report [5], no direct studies of TCPP exposure’s effects on human health have been conducted.

Taking into consideration the detectable levels of TCPP in the environment worldwide, this compound can cause toxic and genotoxic as well as cytotoxic effects in various organisms. Therefore, there is an imperative need for combined genotoxicological studies on the effects of TCPP in organisms from different trophic levels to clearly determine the risks to human health and the environment.

Aquatic pollution is an important environmental issue that faces humanity [23,24,25]. Thus, the potential effects of anthropogenic chemicals such as TCPP on organisms and the environment should be evaluated through a combination of bioassays to fully investigate and determine the risk posed by the studied compound [26]. For this reason, in the present study, the toxic and genotoxic effects of TCPP were investigated using organisms from different trophic levels, including bacteria (*Aliivibrio fischeri*) and green microalgae (*Scenedesmus rubescens*, *Chlorococcum* sp., *Dunaliella tertiolecta, Tisochrysis lutea*) as well as human cells (lymphocytes).

The *Aliivibrio fischeri* bioassay, using the marine bacterium *Aliivibrio fischeri* (formerly known as *Vibrio fischeri*), is a widely used short-term toxicity test. The application of this method is based on the bioluminescent properties of the used bacteria; reductions in the bioluminescence of a bacterium when exposed to chemicals reflects the toxic effect of the tested substances. The *Aliivibrio fischeri* bioassay is used as a standard (eco)toxicological bioassay [27,28].

The use of freshwater and/or marine microalgae has found extensive applications and constitutes an efficient experimental tool for environment contamination assessments and for aquatic ecosystems. For the assessment of TCPP’s toxic effects on freshwater and marine microalgae, the guidelines produced by the Organization for Economic Cooperation and Development (OECD) were followed [29,30].

Over the last few decades, the implementation of the *in vitro* micronucleus assay for the detection of micronuclei (MN) has been extensively applied and proposed as a well-established tool for genotoxicity screening due to its simplicity, rapidity and sensitivity [31,32]. MN formation occurs due to different causative factors such as acentric chromosome fragments or whole chromosomes, as a result of the DNA damage after exposure to a test chemical [26,33]. In the present study, and as part of our efforts to cover the potential effects of TCPP on the genetic material of human cells, the genotoxic and cytotoxic effects of TCPP were evaluated in vitro in cultured human lymphocytes by using the cytokinesis block micronucleus (CBMN) assay. By using all the mentioned bioassays, it is expected that valuable knowledge can be added on the toxic and genotoxic status of the widely used and commercially available TCPP on bacteria, green microalgae and human cells.

## 2. Materials and Methods

### 2.1. Chemicals and Reagents

TCPP (CAS Number:13674-84-5) as a mixture of isomers was purchased from Sigma-Aldrich. Ultrapure water was used for the preparation of the solutions. The microalgae species *Scenedesmus rubescens* (strain SAG 5.95) and *Chlorococcum* sp. (strain SAG 22.83) were purchased from the bank SAG collection of the Gottingen University (Germany). *Dunaliella tertiolecta* (strain CCAP 19/6B) as well as *Tisochrysis lutea* (T. ISO strain CCAP 927, formerly listed as *Isochrysis* sp., *Isochrysis galbana*) were purchased from the Scottish Marine Institute, Oban, Argyll, Scotland. BG-11 medium (Cyanobacteria BG-11 Freshwater Solution) and F/2 medium (Guillard’s (F/2) Marine Water Enrichment Solution) were purchased from Sigma-Aldrich. *Aliivibrio fischeri* bacteria (Microtox^®^ Acute Reagent) and Microtox^®^ Reconstitution Solution were supplied from Modern Water. Sodium chloride, phenol and ZnSO_4_·7H_2_O were obtained from Sigma-Aldrich. For the cell cultures, all the chemicals/reagents reported in our previous work were used [30]. Nylon Syringe Filters (0.22 μm) from Membrane Solutions were also used.

### 2.2. Algal Biotest

Algal bioassays were conducted using the freshwater species *Scenedesmus rubescens* (strain SAG 5.95) and *Chlorococcum sp.* (strain SAG 22.83), as well as the saltwater species *Dunaliella tertiolecta* (CCAP19/6B) and *Tisochrysis lutea* (T. ISO strain CCAP 927), according to the OECD 201 protocol [25]. BG-11 medium (24 ± 1 °C, pH 8.3 ± 0.3) and F/2 without Si (24 ± 1 °C, pH 8.3 ± 0.3, salinity 35%) were used as culture media for fresh and salt-water algal strains, respectively. The experiments were performed by appropriate transfers, under sterile conditions, of stock algal cultures to conical flasks containing BG-11 medium or F/2 medium to maintain the supply of cells (1 × 10^4^ cells mL^−1^) in the logarithmic growth phase (final volume 100 mL). All the cultures were incubated under sterile conditions and continuous illumination (4300 lux). Five different TCPP nominal concentrations (0.5, 1, 10, 20 and 50 μg L^−1^) were tested in duplicate cultures for 72 h under continuous stirring. The cell numbers were determined using a Neubauer hemocytometer and the algae growth (μ) and inhibition (% I) rates were determined at 24, 48 and 72 h of incubation.

### 2.3. Aliivibrio fischeri Bioluminescence Inhibition Test

The ecotoxicity of TCPP was also assessed by the *Aliivibrio fischeri* Bioluminescence Inhibition bioassay using a Microtox Model 500 Toxicity Analyzer from Azur Environmental. The aqueous samples of TCPP were tested in four dilutions in 2% NaCl and incubated for 5 and 15 min. MicrotoxOmni Windows Software was employed for the calculation of the bioluminescence inhibition (%) of each sample and EC_50_ upon 5 and 15 min. Positive (Phenol and ZnSO_4_·7H_2_O) and negative controls (2% NaCl) were also tested, employing the same procedure.

### 2.4. CBMN Assay

The cytokinesis-block micronucleus (CBMN) assay was performed according to the established protocol of our laboratory [34] which is based on the standard procedures of OECD (2016) [33] with minor modifications. Blood samples from two non-smokers (22 and 23 years old) and healthy volunteers were collected. Duplicate cultures were set up for each donor (4 independent cultures in total). The detailed experimental procedure of CBMN assay is depicted in Scheme S1. For the calculation of micronucleus (MN) frequency, 4000 binucleated (BN) cells with preserved cytoplasm were scored in each experimental point according to standard criteria [35,36]. To evaluate the Cytokinesis Block Proliferation Index (CBPI), 2000 cells were analyzed (500 cells per experimental point of each donor) [37]. The calculation of CBPI is given by Equation (1):

CBPI = [M1 + 2 × M2 + 3 × (M3 + M4)]/N(1)

where M1, M2, M3 and M4 correspond to the numbers of cells with one, two, three and four nuclei, while N is the total number of cells.

### 2.5. Statistics

For the determination of IC_50_ values (50% algal growth inhibition), Probit analysis (*p* < 0.05) was performed using *log*-transformed values (IBM SPSS 25 Inc. software package). Levene’s test of equality of error variances was used to check the homogeneity of variance, whereas significant differences in the algae number (mL^−1^ × 10^4^) were assessed by post-hoc multiple comparison tests (Bonferroni test, *p* < 0.05, ANOVA). Using Microtox assay the bioluminescence inhibition (%) of each sample, as well as the effective concentration of the TCPP that reduced the bioluminescence by 50% (EC_50_), were calculated using MicrotoxOmni Windows Software. Regarding the CBMN assay, the G-test was used for MN data analysis, whereas the CBPI data set was analyzed using the Chi-square (×2) test.

### 2.6. Ethic Statement

The study was approved by the Research Ethics Committee of the University of Patras (Ref. No. 7682/6 June 2021). Participants used as blood donors signed informed consent forms to say that they were not exposed to radiation, drug treatments or any viral infection in the recent past.

## 3. Results and Discussion

### 3.1. Genotoxicity Evaluation Using the CBMN Assay

Anthropogenic chemicals with extensive use in a plethora of applications—such as TCPP—tend to accumulate in ecosystems, posing a risk to living organisms. The undesired effects range from potential toxicological alterations of micro-organisms to genotoxicological damages in higher organisms, including humans. Species from different trophic levels have been used in many studies as bioindicators to evaluate the potential toxicological effects of several environmental contaminants [26,30]. The implementation of various toxicity bioassays in aquatic micro-organisms, in combination with the application of an in vitro genotoxicity assay—such as the CBMN assay in human cells—followed herein, provides a comprehensive picture of the potential effects of widely used and commercially available chemicals, such as TCPP product. 

#### Genotoxicological Effects on Human Lymphocytes

**Figure 1:**
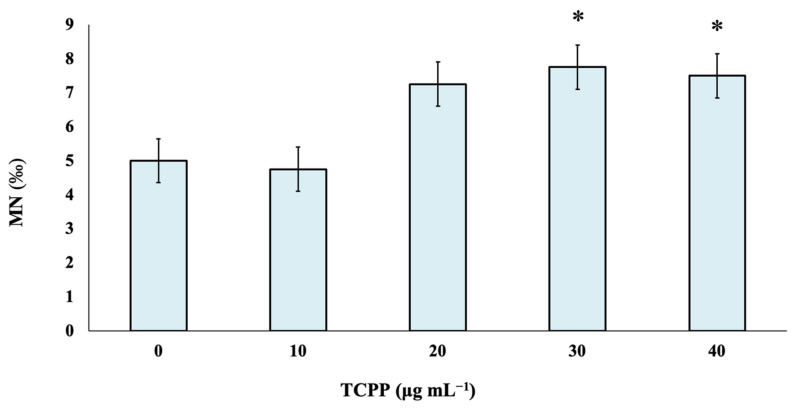
Genotoxic effects of TCPP in human lymphocytes. MN frequencies are expressed as mean frequencies (‰) ± standard error. * *p* < 0.05 (significant differences compared to the control value, G-test).

TCPP was studied at four different nominal concentrations—namely, 10, 20, 30 and 40 μg mL^−1^—to identify its potential risk of inducing genotoxic effects in cultured human lymphocytes. As observed, there are no statistically significant differences between control and TCPP-treated cultures at the lowest tested nominal concentrations of 10 and 20 μg mL^−1^. Statistically significant differences in MN frequencies in comparison with the control were recorded at TCPP nominal concentrations of 30 and 40 μg mL^−1^ (Figure 1).

**Figure 2:**
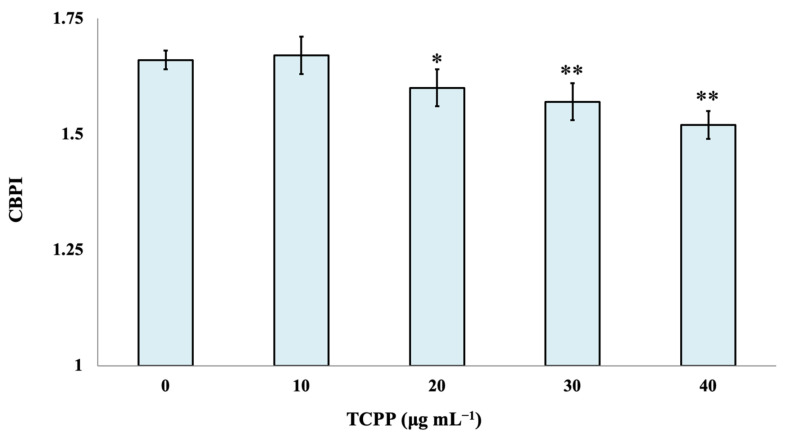
Cytotoxic effects of TCPP in human lymphocytes. CBPI values are expressed as mean frequencies (‰) ± standard error. * *p* < 0.05, ** *p* < 0.001 (significant differences compared to the control value, χ2-test).

The cytotoxic effect of TCPP was evaluated by the determination of the CBPI index. Statistically significant differences in CBPI were observed between control cultures and the examined nominal concentrations of 20, 30 and 40 μg mL^−1^ of TCPP. TCPP showed enhanced cytotoxic effects (*p* < 0.001) at the highest examined concentrations of 30 and 40 μg mL^−1^ (Figure 2).

Experimental animal studies reported that TCPP causes developmental toxicity and affects the immune response and metabolism of chicken embryos [38,39].

The exposure to TCPP of zebrafish larvae, as a model for vertebrate embryogenesis, provokes neurobehavioral toxicity but does not cause developmental malformations [40,41]. Up to now, there have been only a few studies focusing on the effects of TCPP in mammalian cell lines. Exposure to TCPP decreased the number of neurocytes and altered the phenotypic neurodifferentiation of a PC12 cell line from rats [42]. Besides this, it has been reported that TCPP affects reproductive hormones in human cell lines [43] and displays agonistic pregnane X receptor activity in simian kidney cells [44].

In a study performed by Föllmann and Wober (2006) [21], the genotoxicological effects of TCPP in V79 (hamster fibroblasts) cells and its mutagenic potential in different *Salmonella* strains were evaluated. TCPP showed cytotoxicity above 1 mM in the presence of S9 in V79 cells but did not induce DNA-strand breaks in the alkaline version of the Comet assay in the presence and absence of a metabolic activation system (S9-mix). No mutagenic effects were observed for TCPP in eight *Salmonella* strains using concentrations of up to 1 mM in the presence and absence of S9. On the other hand, a recent study reported that TCPP induced genotoxic and apoptotic effects in human umbilical vein endothelial cells (HUVECs) and may affect the human vascular system [22]. In a recent report [5], no evidence of developmental toxicity caused by TCPP was observed in Sprague Dawley rats in the absence of overt maternal toxicity. Concerning the possible genotoxicological profile of TCPP in human lymphocytes cultures, it was observed that only the highest tested nominal concentrations (30 and 40 μg mL^−1^) induced a marginally genotoxic effect and a significant cytotoxic effect under the specific experimental conditions. Our findings agree with the results of the aforementioned studies focused on the genotoxicological activities of TCPP in different cell lines and organisms.

The maximum average concentration of TCPP worldwide was detected in Chinese water resources, at around 67 μg mL^−1^, as stated in a very recent review where the global distribution of OPEs in aquatic systems was analyzed. Moreover, carcinogenic risks were not reported for OPEs and potable water in the examined countries and the averages are above the limit for only one country (China) [45]. The concentrations of TCPP used in the present study (10 up to 40 μg mL^−1^) are below or comparable to the maximum average detected concentration of TCPP in China. Our findings and the available literature data suggest a potential risk for the utilization of TCPP, taking into consideration the observed *in vitro* genotoxicological effects in human lymphocytes as well as in various human cell lines.

### 3.2. Ecotoxicity Assessment

The continuous release of OPEs into aquatic systems and their potential toxic effects on living organisms have caused a growing concern about their impact on the environment. Algae, crustaceans and fish are representative aquatic organisms that have been used as bioindicators for the environmental risk assessment of pollutants [46,47], including OPEs [45,48,49,50,51]. Based on literature data, OPEs can cause toxic effects to all of these organisms [52]. In the present study, the possible toxic effects of TCPP in fresh- and marine- water algal species, as well as in bacteria, were evaluated in detail.

#### 3.2.1. Effects of TCPP on Freshwater and Saltwater Algal Species

**Figure 3:**
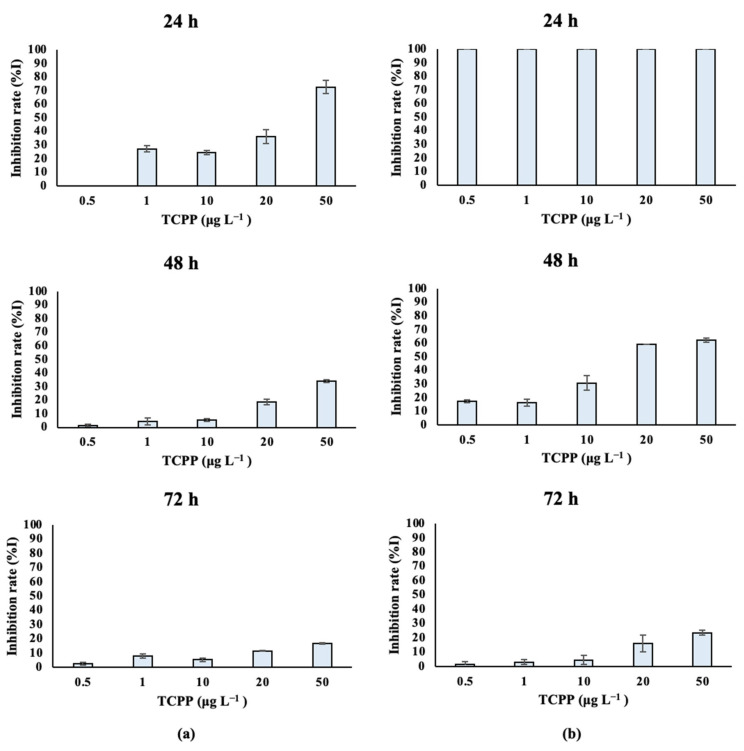
Inhibition of (**a**) *Scenedesmus rubescens* and (**b**) *Chlorococcum* sp. growth rate (%I) after exposure to TCPP for 24, 48 and 72 h.

Fresh-water algal species treated with TCPP showed different growth rates over time, as depicted in Table S2A and Figure 3. Specifically, *Scenedesmus rubescens* treated with 1, 10, 20 and 50 μg L^−1^ of TCPP for 24 h showed significantly lower growth rates compared to control cultures (Figure 3a). The same effect was also observed at 48 h for the highest concentrations of TCPP (20 and 50 μg L^−1^). In contrast, lower effects on growth rates were observed after the exposure of *Scenedesmus rubescens* for 72 h to TCPP, as confirmed by the increase in IC_50_ values over time (Table 1). In the case of *Chlorococcum* sp. (Table S2B, Figure 3b), a total inhibition (%I) of algae growth occurred at 24 h of exposure for all nominal concentrations of TCPP tested (0.5, 1, 10, 20 and 50 μg L^−1^). At 48 h, significantly lower growth rates and higher inhibition (%I) of algae growth were also recorded as compared to the control—specifically at the highest nominal concentrations (20 and 50 μg L^−1^). Finally, inhibition rates after algae exposure for 72 h revealed that TCPP still inhibited the growth rate of *Chlorococcum sp.,* while the %I stayed higher at the highest tested concentrations (20 and 50 μg L^−1^). IC_50_ values (Table 1) were in agreement with inhibition rates; it was observed that at 48 h with high inhibition, the IC_50_ concentration was 0.019 mg L^−1^, while at 72 h IC_50_ it remained low (0.73 mg L^−1^).

**Figure 4:**
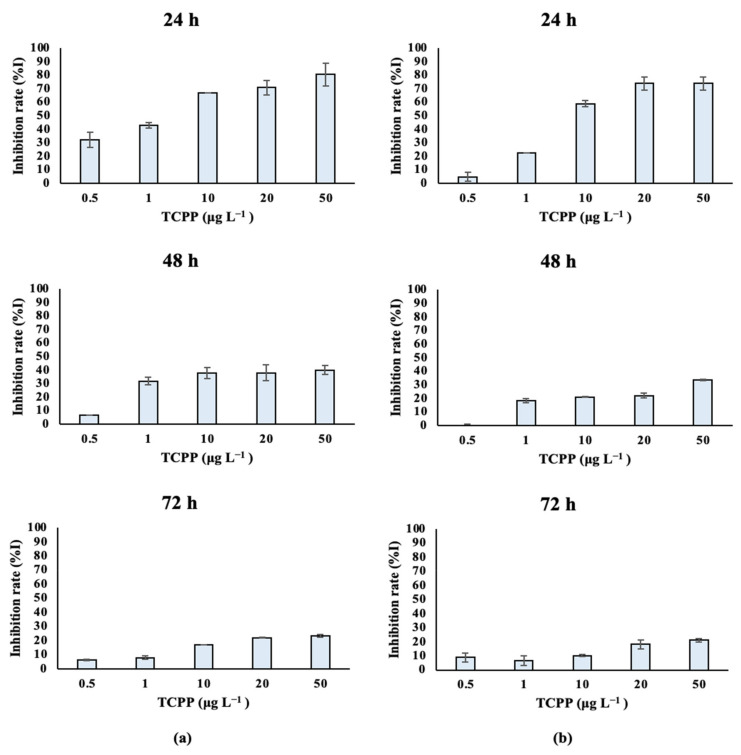
Inhibition of (**a**) *Dunaliella tertiolecta* and (**b**) *Tisochrysis lutea* growth rate (%I) after exposure to TCPP for 24, 48 and 72 h.

Marine algal species treated with TCPP showed similar growth rates over time. *Dunaliella tertiolecta* cultures (Table S3A, Figure 4a) treated with different nominal concentrations of TCPP (0.5, 1, 10, 20 and 50 μg L^−1^) showed high growth inhibition at the 24 and 48 h at all tested nominal concentrations, with IC_50_ values of 0.002 and 0.114 mg L^−1^ respectively. After 72 h of treatment, the inhibition rate was still high—especially for the nominal concentrations of 10, 20 and 50 μg L^−1^—and the IC_50_ value was 1.596 mg L^−1^. Similarly, lower growth rates and algal density were observed for *Tisochrysis lutea* (Table S3B; Figure 4b) treated with TCPP (0.5, 1, 10, 20 and 50 μg L^−1^) over time (24–48–72 h), showing a dose–response relationship. IC_50_ values (Table 1) are in accordance with the low growth rate of *Tisochrysis lutea* after treatment with TCPP. IC_50_ values equal to 0.007, 0.354 and 21.52 mg L^−1^ were determined at 24, 48 and 72 h, respectively (Table 1).

After exposure to TCPP, the studied algae were affected in terms of their growth and algal cell density. A significant inhibition of the growth rate during the first 24 h was observed for all the tested algal species.

However, differences among the algal species’ sensitivities were observed at higher exposure times—especially at 72 h. The differences in their IC_50_ values can be correlated with the different sensitivities of the microalgal species [53]. In the case of the freshwater species, the most sensitive was found to be *Chlorococcum* sp. On the other hand, *Scenedesmus rubescens* was found to be the most resistant organism, and a significant increase in its IC_50_ values was observed over time. In the case of saltwater species, it was observed that both algal species *Dunaliella tertiolecta* and *Tisochrysis lutea* were significantly affected after exposure to TCPP, which was also confirmed by the low IC_50_ values. The most sensitive organism, by a small margin, was found to be *Dunaliella tertiolecta*, with a low recovery of the growth rate over time (72 h). Growth inhibition in microalgae reflects the toxic effects of different pollutants on microalgae such as TCPP, affecting their physiological function [54]. Niu et al. (2019) [52] mentioned that the IC_50_ value for the species *Scenedesmus subspicatus* was 45 mg L^−1^ after exposure to TCPP for 72 h, which is consistent with our findings for the *Scenedesmus rubescens*. According to the United Nations, compounds are classified based on ecotoxicity values as follows [55]:

(i) highly toxic: EC(IC)_50_ ≤ 1 mg L^−1^;
(ii) toxic: 1 mg L^−1^ < EC(IC)_50_ ≤ 10 mg L^−1^;
(iii) harmful to aquatic organisms: 10 mg L^−1^ < EC (IC)_50_ ≤ 100 mg L^−1^.

Based on the IC_50_ values calculated in the present study (Table 1), TCPP is highly toxic to *Chlorococcum* sp., toxic to *Dunaliella tertiolecta* and harmful to *Scenedesmus rubescens* and *Tisochrysis lutea*.

In the present study, differences between the organisms regarding their response to the specific pollutant were observed. Differences in cell sizes, the presence/absence of a cell wall and the ability of each species to absorb and eliminate pollutants can be considered to justify the observed responses to the tested algae [56]. Furthermore, the adaptability of all four studied species was observed against TCPP over time. Specifically, in the first 24 and 48 h high inhibition rates occurred with the percentage of TCPP, but at 72 h, the inhibition rates were significantly reduced. Microalgal adaptation mechanisms against certain stress factors, such as toxic substances, could be considered to explain the observed trends [57]; these mechanisms involve the secretion of high molecular-weight natural polymers known as extracellular polymeric substances (EPS) out of the cell. In microalgae, EPS are synthesized in the Golgi apparatus and protect the cells from harsh environmental conditions, creating a slimy coating around them [58].

#### 3.2.2. *Aliivibrio fischeri* Bioluminescence Inhibition Test

**Figure 5:**
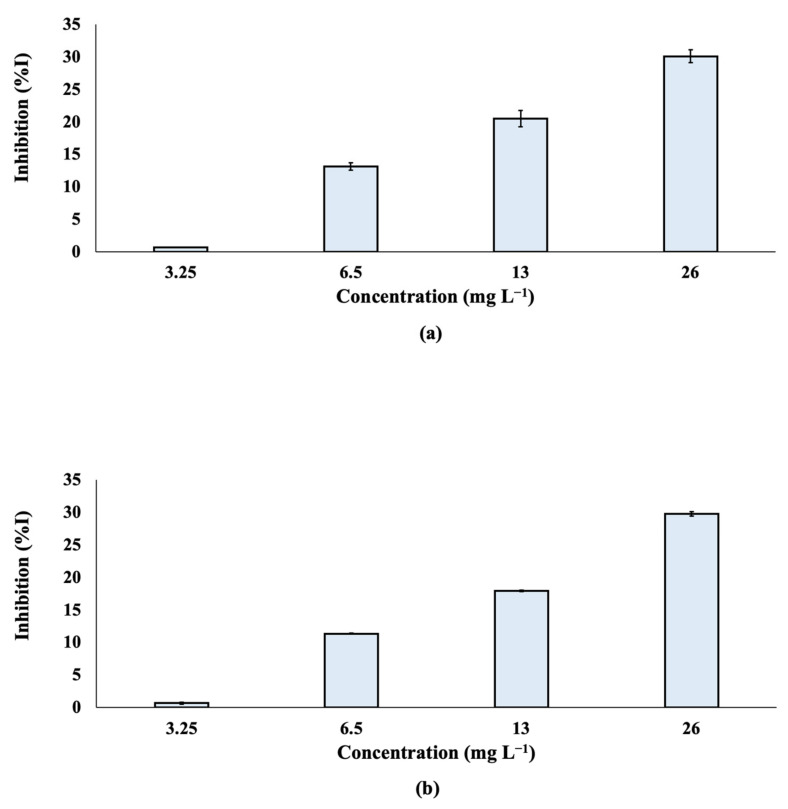
% Inhibition of bioluminescence of *Aliivibrio fischeri* after (**a**) 5 min and (**b**) 15 min of exposure to TCPP.

A Microtox^®^ assay was performed and the % effect of inhibition was determined after the exposure of *Alivibrio fischeri* to TCPP at different nominal concentrations (3.25, 6.5, 13 and 26 mg L^−1^; Figure 5). The effective concentration of TCPP that reduced bioluminescence by 50% (EC_50_) was also calculated (Figure S1) and found to be 71.5 mg L^−1^ for both 5 and 15 min of exposure. According to the results, the % inhibition of TCPP was not significantly differentiated at 5 and 15 min.

Based on the results, TCPP can be classified as a “harmful” compound to *Aliivibrio fischeri*. The obtained results agree with the available data in the literature. A value of LC_50_ equal to 81 mg L^−1^ has been reported for *Daphnia magna* [59]. Similar results were found in an earlier publication for the species *Daphnia magna*, with an LC_50_ value of 91 mg L^−1^. Exposure of *Mytilus galloprovincialis* to 10 and 100 nmol L^−1^ of TCPP for a period of 42 days enhances reactive oxygen species (ROS) production, oxidative stress and apoptosis [60]. Moreover, TCPP has been found to affect the muscles and nervous system development of the ascidian *Ciona intestinalis* [61].

## 4. Conclusions

In the present study, the implementation of a battery of toxicity bioassays in aquatic micro-organisms in combination with the application of an in vitro genotoxicity assay in human cells was performed to evaluate the potential effects of TCPP in the environment and humans. The potential risk of TCPP (10, 20, 30 and 40 μg mL^−1^) inducing genotoxic and cytotoxic effects in cultured human lymphocytes was evaluated using a CBMN assay. Only the highest nominal concentrations (30 and 40 μg mL^−1^) induced marginally significant MN frequencies as well as cytotoxic effects. The effects of TCPP (0.5 1, 10, 20 and 50 μg L^−1^) in freshwater and marine algal species were also evaluated. Reduced growth rates for all tested microalgae species were observed after exposure for 24 h, reaching a 100% inhibition of the growth rate in some cases. Among the freshwater species, *Chlorococcum* sp. was found to be the most sensitive. Both tested marine microalgae species (*Dunaliella tertiolecta* and *Tisochrysis lutea*) were significantly affected after exposure to TCPP. The effect of the tested flame retardant was also estimated against the bacteria *Aliivibrio fischeri*. According to the categories established by the United Nations, TCPP can be considered as highly toxic to *Chlorococcum* sp., toxic to *Dunaliella tertiolecta* and harmful to *Scenedesmus rubescens*, *Tisochrysis lutea* and *Aliivibrio fischeri*. These findings suggest that the wide utilization of TCPP and its consequent release into the environment could have adverse effects on aquatic organisms and humans.

## Figures and Tables

**Table 1:** TCPP inhibitory concentrations (24, 48 and 72 h IC_50_ values) for *Scenedesmus rubescens*, *Chlorococcum sp.*, *Dunaliella tertiolecta*, and *Tisochrysis lutea*.

	IC_50_ (mg L^−1^)			
Exposure Period (h)	*Scenedesmus rubescens*	*Chlorococcum sp.*	*Dunaliella tertiolecta*	*Tisochrysis lutea*
24	0.026	ND	0.002	0.007
48	0.233	0.019	0.114	0.354
72	58.420	0.73	1.596	21.52

## Data Availability

Data are contained within the article.

## Supplementary Materials

The following supporting information can be downloaded at: https://www.mdpi.com/article/10.3390/toxics10120736/s1. Table S1, Structures of TCPP isomers [5]; Table S2,TCPP effects of (A) *Scenedesmus rubescens* and (B) *Chlorococcum sp.* on cell number (cells/mL × 10^4^) and algal growth rate (μ values in the parenthesis); Table S3, TCPP effects of (A) *Dunaliella tertiolecta* and (B) *Tisochrysis lutea* on cell number (cells/mL × 10^4^) and algal growth rate (μ values in the parenthesis); Scheme S1, Experimental procedure of CBMN assay; Figure S1, EC_50_ (mg L^−1^) of TCPP in *Aliivibrio fischeri*.
