# Supplementary material for: Genotoxic and Toxic Effects of The Flame Retardant Tris(Chloropropyl) Phosphate (TCPP) in Human Lymphocytes, Microalgae and Bacteria
Source: Toxics. 2022 Nov 28;10(12):736. doi: 10.3390/toxics10120736 (PMC9782401; doi:10.3390/toxics10120736)
Supplement: Supplementary file 1 [file toxics-10-00736-s001.zip › toxics-2014730-supplementary.pdf]

toxics

MDPI

Supplementary Materials: Genotoxic and Toxic Effects of The Flame Retardant Tris(chloropropyl) Phosphate (TCPP) in Human Lymphocytes, Microalgae and Bacteria

Maria Antonopoulou, Dimitris Vlastos, Margarita Dormousoglou, Spyridon Bouras, Maria Varela-Athanasatou and Irene-Eleni Bekakou

Table S1. Structures of TCPP isomers.

Isomer	Chemical Structure
Tris(1-chloro-2-propyl) phosphate	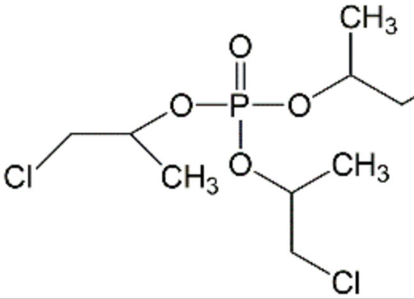
Bis(2-chloro-1-methylethyl)-2-chloropropyl phosphate	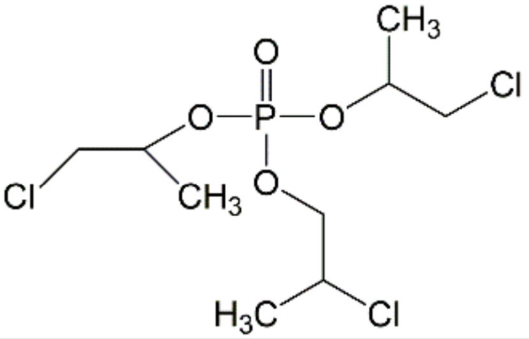
Bis(2-chloropropyl) 2-chloroisopropyl phosphate	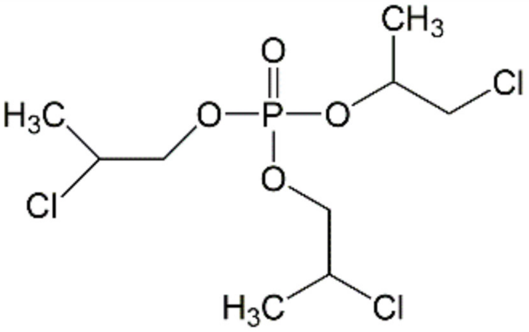
Tris(2-chloropropyl) phosphate	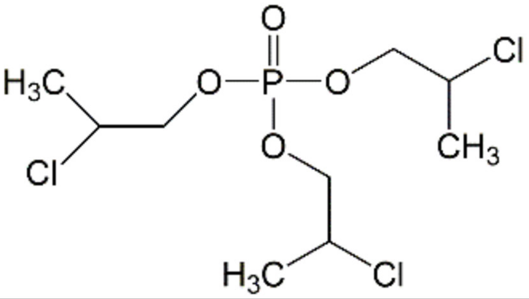

Table S2. TCPP effects of (A) *Scenedesmus rubescens* and (B) *Chlorococcum* sp. on cell number (cells/mL x 10^4) and algal growth rate (μ values in the parenthesis).

	Incubation time (h)		
	24	48	72
	(A) *Scenedesmus rubescens*		
Control	2.63 ± 0.18 (0.93 ± 0.07)	8.71 ± 0.29 (1.08 ± 0.02)	21.15 ± 0.68 (1.02 ± 0.01)
	TCPP ($\mu g\ L^{-1}$)		
0.5	2.56 ± 0.09 (0.94 ± 0.03)^{abcd}	8.42 ± 0.12 (1.07 ± 0.01)^{abc}	19.50 ± 0.53 (0.99 ± 0.01)^{*abcd}
1	1.97 ± 0.04 (0.68 ± 0.02)^{*ae}	7.94 ± 0.44 (1.04 ± 0.03)^{*de}	16.69 ± 0.80 (0.94 ± 0.02)^{*aeg}
10	2.02 ± 0.03 (0.70 ± 0.01)^{*bf}	7.75 ± 0.18 (1.02 ± 0.01)^{*afg}	17.98 ± 0.68 (0.96 ± 0.01)^{*behi}
20	1.81 ± 0.09 (0.59 ± 0.05)^{*cg}	5.80 ± 0.25 (0.88 ± 0.02)^{*bdfh}	14.88 ± 0.18 (0.90 ± 0.00)^{*cfhj}
50	1.29 ± 0.06 (0.26 ± 0.05)^{*defg}	4.19 ± 0.09 (0.72 ± 0.01)^{*cegh}	12.63 ± 0.18 (0.85 ± 0.00)^{*dgij}
	(B) *Chlorococcum sp.*		
Control	1.19 ± 0.09 (0.19 ± 0.06)	6.29 ± 0.29 (0.92 ± 0.02)	8.19 ±0.27 (0.70 ± 0.01)
	TCPP ($\mu g\ L^{-1}$)		
0.5	0.98 ± 0.03 (0.00 ± 0.00)^{*abc}	4.56 ± 0.09 (0.76 ± 0.01)^{*abc}	7.92 ± 0.26 (0.69 ± 0.01)^{ab}
1	0.88 ± 0.00 (0.00± 0.00)^{*def}	4.67 ± 0.24 (0.77 ± 0.03)^{*def}	7.69 ± 0.27 (0.68 ± 0.01)^{cd}
10	0.46 ± 0.06 (0.00± 0.00)^{*ad}	3.58 ± 0.35 (0.64 ± 0.05)^{*adgh}	7.48 ± 0.50 (0.67 ± 0.02)^{ef}
20	0.44 ± 0.03 (0.00± 0.00)^{*be}	2.13 ± 0.00 (0.38 ± 0.00)^{*beg}	5.88 ± 0.71 (0.59 ± 0.04 ^{*ace}
50	0.35 ± 0.03 (0.00± 0.00)^{*cf}	2.00 ± 0.06 (0.35 ± 0.01)^{*cfh}	5.00 ± 0.18 (0.54 ± 0.01)^{*bdf}

Table S3. TCPP effects of (A) *Dunaliella tertiolecta* and (B) *Tisochrysis lutea* on cell number (cells/mL x 10^4) and algal growth rate (μ values in the parenthesis).

	Incubation time (h)		
	24	48	72
	(A) *Dunaliella tertiolecta*		
Control	2.40 ± 0.01 (0.87 ± 0.06)	3.73 ± 0.21 (0.65 ± 0.02)	8.94 ± 0.27 (0.73 ± 0.01)
	TCPP ($\mu g\ L^{-1}$)		
0.5	1.81 ± 0.09 (0.59 ± 0.05)^{*abcd}	3.38 ± 0.00 (0.61 ± 0.00)^{abcd}	7.81 ± 0.09 (0.69 ± 0.00)^{*abcd}
1	1.65 ± 0.03 (0.50 ± 0.02)^{*aefg}	2.44 ± 0.09 (0.45 ± 0.02)^{*a}	7.50 ± 0.18 (0.67 ± 0.01)^{*aefg}
10	1.33 ± 0.00 (0.29 ± 0.00)^{*beh}	2.25 ± 0.12 (0.41 ± 0.03)^{*b}	6.13 ± 0.00 (0.60 ± 0.00)^{*behi}
20	1.29 ± 0.06 (0.26 ± 0.05)^{*cf}	2.25 ± 0.18 (0.40 ± 0.04)^{*c}	5.52 ± 0.03 (0.57 ± 0.00)^{*cfh}
50	1.19 ± 0.09 (0.17 ± 0.07)^{*dgh}	2.19 ± 0.09 (0.39 ± 0.02)^{*d}	5.33 ± 0.12 (0.56 ± 0.01)^{*dgi}
	(B) *Tisochrysis lutea*		
Control	1.69 ± 0.03 (0.52 ± 0.02)	7.63 ± 0.18 (1.02 ± 0.01)	32.67 ± 0.47 (1.16 ± 0.00)
	TCPP ($\mu g\ L^{-1}$)		
0.5	1.65 ± 0.03 (0.50 ± 0.02)^{abcd}	7.65 ± 0.15 (1.02 ± 0.01)^{abcd}	24.06 ± 2.56 (1.06 ± 0.04)^{*ab}
1	1.50 ± 0.00 (0.41 ± 0.00)^{*aefg}	5.25 ± 0.18 (0.83 ± 0.02)^{*aef}	26.17 ± 3.06 (1.09 ± 0.04)^{*cd}
10	1.24 ± 0.01 (0.22 ± 0.01)^{*behi}	4.98 ± 0.03 (0.80 ± 0.00)^{*bg}	22.88 ± 0.53 (1.04 ± 0.01)^{*ef}
20	1.15 ± 0.03 (0.14 ± 0.03)^{*cfh}	4.88 ± 0.18 (0.79 ± 0.02)^{*ceh}	17.40 ± 1.97 (0.95 ± 0.04)^{*ace}
50	1.15 ± 0.03 (0.14 ± 0.03)^{*dgi}	3.85 ± 0.03 (0.67 ± 0.00)^{*dfgh}	15.65 ± 0.74 (0.92 ± 0.02)^{*bdf}

Values with * in each column differ significantly from control (One-way Anova, $p< 0.05$). Values in each column that share the same letter are significantly different from each other.

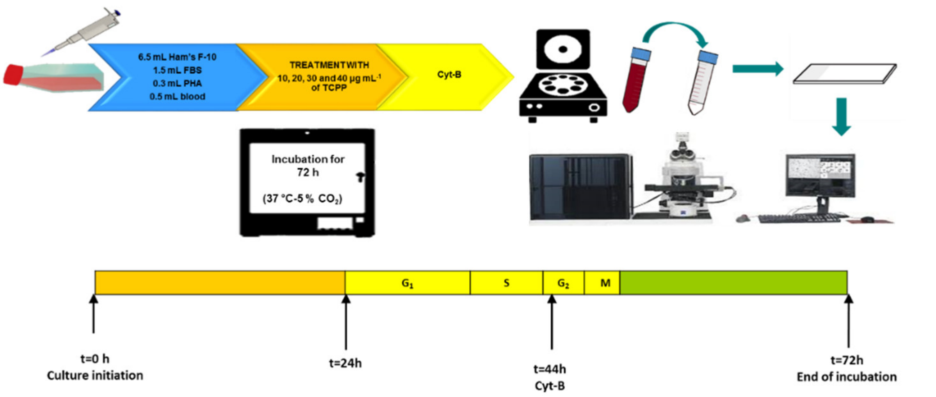

Scheme S1. Experimental procedure of CBMN assay.

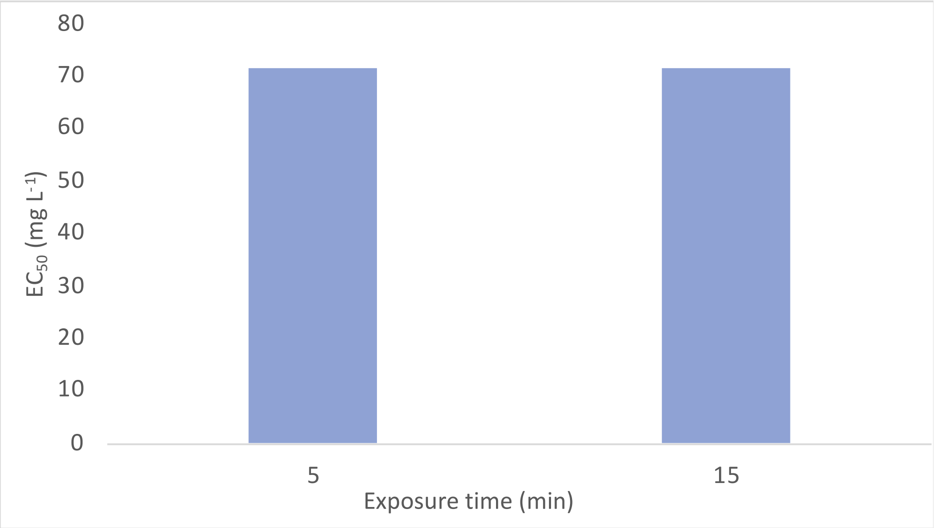

Figure S1. EC_{50} (mg L^{-1}) of TCPP in *Aliivibrio fischeri*.
